# Improving energy autonomy of positive energy districts using multi-agent deep reinforcement learning

**DOI:** 10.1038/s41598-025-12554-x

**Published:** 2025-07-30

**Authors:** Jernej Hribar, Mihael Mohorčič, Andrej Čampa

**Affiliations:** 1 Jozef Stefan Institute, Jamova cesta 39, 1000 Ljubljana, Slovenia; 2 Comsensus, Brezje pri Dobu 8a, 1233 Dob, Slovenia

**Keywords:** Electrical and electronic engineering, Information technology

## Abstract

In recent years, Positive Energy Districts (PEDs) have emerged at the forefront of urban innovation, rapidly transforming communities by integrating shared Energy Storage Systems (ESS) and Electric Vehicles (EVs) to redefine the future of sustainable communities. However, energy management in such communities remains extremely challenging due to the dynamic nature of EV availability, unpredictable renewable energy generation, and the necessity to maintain user comfort while optimizing energy use. Overcoming these challenges is critical for enabling PEDs to achieve carbon neutrality, reduce costs, and improve energy sharing. In addition, Vehicle-to-Grid (V2G) technology and shared ESS offer unique opportunities to optimize energy consumption and facilitate access to the open energy market, but fully exploiting their potential requires advanced strategies such as Deep Reinforcement Learning (DRL). To address these needs, this work proposes a novel Community Multi-Agent Deep Reinforcement Learning Vehicle-to-Grid (CoMAD V2G) solution based on Multi-Agent Reinforcement Learning (MARL), which enhances the utilization of community-generated energy and increases community autonomy by controlling the charging and discharging cycles of V2G-enabled EVs. Real data on household consumption, solar energy production, EV dynamics, and electricity prices are used to evaluate and verify the effectiveness of the proposed solution in a realistic environment. Under these conditions, the proposed solution achieves improved energy exchange with the external grid on an annual basis, a result not attained with comparable conventional heuristic or alternative learning-based approaches for the community under consideration. Furthermore, the solution reduces household electricity costs by up to 25%, highlighting its potential to deliver significant economic and sustainability benefits for PEDs.

## Introduction

Over the past two decades, the emergence of Positive Energy Districts (PEDs)^1^ has marked a transformative shift in urban energy systems, emphasizing the integration of renewable energy sources, energy efficiency, and smart technologies to create energy-positive communities. Often these communities have a community battery, which, as the name suggests, is a storage solution for multiple households in an area. It stores excess energy generated by photovoltaics (PV) or other renewable sources whenever it is available, e.g., during the day, and provides energy to the community when energy from renewable sources is not available, e.g., during the night. These batteries also provide an opportunity to further enhance energy sharing and lead communities and cities towards carbon neutrality^2^. In addition, by sharing an Energy Storage System (ESS), communities can reduce deployment and maintenance costs while participating in the open energy market, an option not typically available to an individual household, leading to further benefits that such a community can offer.

Electric Vehicless (EVs) have become an important element in PED, bringing flexibility and complexity to operations. Through Vehicle-to-grid (V2G) technology, EVs serve not only as a means of transportation for residents, but also as an additional mobile ESS. Consequently, the community is able to improve its control over energy generated from renewable sources such as PV and store it for later use or share it with the wider community. Unfortunately, the integration of V2G-enabled EVs also introduces significant dynamics into the system. For example, the presence of vehicles is not constant, and their state of charge upon arrival in the community is often unpredictable. The charging or discharging of these vehicles must therefore be carefully managed to ensure a non-intrusive approach that maintains user comfort and does not interfere with the primary use of the vehicles. Despite these challenges, V2G technology offers a unique opportunity for PED, where combining household energy resources with shared ESS and V2G-enabled EVs also introduces significant dynamics into offers benefits to users, such as lower costs. In such a system, existing methods, e.g., traditional heuristic rule-based approaches, static optimization techniques, centralized single agent control, etc., lack adaptive capabilities and cannot dynamically respond to rapidly changing energy demands, fluctuating renewable energy generation, and varying EV availability. This highlights the necessity of an intelligent and collaborative deep Multi-Agent Reinforcement Learning(MARL)-based solution that can learn optimal strategies for charging or discharging V2G-enabled EVs to maximize the energy gains of the community while ensuring minimal impact on an individual user.

Deep Reinforcement Learning (DRL) has already demonstrated its potential in a variety of applications related to the energy management of buildings^3^, from optimizing the heating and cooling systems in a building to participating in the energy markets^4^. This paper adopts DRL paradigm and employs its extension, MARL, which is particularly well suited for complex and dynamic environments such as PEDs^5^, where multiple agents that control households, V2G units, or shared ESS need to make joint decisions to maximize the overall performance of the system. In such a system as considered in this work, the availability of resources such as V2G batteries fluctuates depending on user behavior and grid conditions, and sharing an ESS adds another layer of interdependence. While a rule-based solution can help with basic energy allocation, it is not sufficiently adaptive to respond to changing circumstances such as fluctuating energy demand, electricity market prices and renewable energy generation. A MARL-based approach, on the other hand, can learn optimal strategies through interaction of intelligent agents, enabling a “zero-touch” solution that requires minimal user intervention while maintaining comfort and achieving efficiency.

This paper focuses on a community with a shared ESS, in which each household has a PV system and a V2G-enabled EV. In summary, this work makes the following contributions:

- **Proposes Community Multi-Agent Deep reinforcement learning Vehicle-to-Grid (CoMAD V2G), a novel MARL-based approach that uniquely combines decentralized multi-agent decision making with collaborative learning at the community level**. In contrast to existing methods, CoMAD V2G enables each household’s agents to learn both individual and collective strategies for optimal charging and discharging of V2G-enabled EVs, taking into account both user convenience and the state of a shared energy storage system. This collaborative learning mechanism explicitly optimizes community-level goals, such as minimizing aggregate energy costs and maximizing energy autonomy, while respecting user-level constraints.
- **CoMAD V2G is implemented and validated in a realistic, region-specific simulation environment for Slovenia**. The experimental setup combines several real data sources and open source tools including LoadProfileGenerator^6^ for generating household consumption patterns, open data on solar energy production^7^, *emobpy*^8^ for modeling V2G dynamics and real-time electricity prices^9^. This integrated framework ensures the practical relevance and adaptability of the proposed solution in a realistic environment.
- **CoMAD V2G is evaluated and compared with both the standard heuristic baseline solutions, referred to as immediate and night mode, and an alternative MARL approach referred to as individual DRL mode**. Results show that CoMAD V2G achieves overall superior performance. It improves PED energy autonomy by reducing annual grid imports by up to 350kWh per household compared to heuristic methods and by up to 100kWh compared to another MARL-based method. In addition, the proposed approach reduces household electricity costs by up to 25% while maintaining the comfort level of EV users, i.e. not affecting their habits and daily routines.

In addition, CoMAD V2G emphasizes privacy by not sharing sensitive user data, making it a non-intrusive, privacy-preserving, and scalable option for energy management in communities aiming for net-zero emissions. By intelligently deciding when to charge, discharge or purchase electricity, the proposed CoMAD V2G solution helps these communities thrive with minimal intervention from individual users, encouraging collaboration and ensuring the long-term sustainability of the energy system.

## Related work

The benefits of sharing ESS in a community have been analyzed in multiple studies. For example, Roberts *et al.*^10^ analyze ESS deployment in an apartment complex and show that even a simple management strategy, when paired with PV, can increase self-sufficiency and self-consumption while reducing deployment costs for individual households. Similarly, Keck and Lenzen^11^ illustrate how shared ESS enables new business models, including the possibility for a community to act as a single entity,an approach also considered by the proposed solution. Their analysis further demonstrates that shared energy storage can significantly reduce the load on the external grid. This is also confirmed by Berg *et al.*^12^, who focus on the role of shared storage in supporting the Distribution System Operator (DSO). Finally, Parra *et al.*^13^ highlight that community ESS is most cost-effective when its capacity allows full discharge during peak hours. Building on these findings, a solution based on DRL is proposed, capable of adapting and learning to fully maximize the benefits of a shared ESS.

The control of ESS has been extensively studied in the past. For example, Zhang *et al.*^14^ conducted a comparative analysis of ESS-sharing schemes in an industrial park, demonstrating that centralized ESS achieves the lowest operating cost and highest utilization. Erdinç *et al.*^15^ proposed a Mixed-Integer Linear Programming (MILP) framework for energy management in systems integrating PV and ESS. Similarly, MILP was applied by Alnaser *et al.*^16^ to optimize battery scheduling for maximizing energy self-sufficiency in a community while providing short-term operating reserve services to the DSO, ensuring compliance with distribution network constraints. Samadi *et al.*^17^ introduced a heuristic boundary selection approach to enhance the financial and operational effectiveness of V2G charging in residential complexes by improving scheduling within the community. Another strategy, proposed by Tian *et al.*^18^, optimizes the V2G power control strategy based on time-of-use pricing and load cost optimization, leveraging EVs as mobile energy storage units to enhance grid stability and efficiency. In contrast to these centralized or static optimization methods, the proposed solution is distributed and enables decision-making at the individual household level regarding V2G charging scheduling. CoMAD V2G dynamically adapts to environmental changes using DRL, minimizing costs for residents while offering greater flexibility and scalability.

The proposed solution also builds on existing DRL-based solutions. However, the specific objective of CoMAD V2G is to design a V2G charging policy using MARL, without controlling household energy consumption, thereby preserving user comfort and habits, while also integrating a shared ESS. For example, V2G charging management using Reinforcement Learning (RL) has been studied by Hao *et al.*^19^; however, their focus was limited to individual vehicle charging, without considering the dynamics introduced by a community or shared battery. Nakabi *et al.*^20^ benchmarked several DRL approaches for managing energy loads in systems with shared batteries and renewable generation, such as wind turbines. Additionally, Saini *et al.*^21^ proposed a multi-agent-based cloud energy storage framework for residential communities, focusing on distributed control and cost reduction in community storage systems. Baberwal *et al.*^22^ addressed energy management in residential PV-battery systems using Q-learning, targeting optimal energy consumption and battery utilization for individual households. In constrast, the proposed CoMAD V2G solution focuses on policy design for community-shared storage and coordinated charging. Furthermore, CoMAD V2G does not control household loads but instead adopts a less intrusive strategy by designing a V2G charging policy within a community utilizing a shared ESS. Additionally, DRL has been employed by Pokorn *et al.*^23^ to reduce energy costs for individual households through smart charging and discharging policies for household ESSs. The proposed solution extends this concept to a community-based approach. Similarly, Kim *et al.*^24^ applied RL to minimize costs in systems with dynamic pricing by adapting energy consumption scheduling.

## Community with shared ESS

**Fig. 1:**
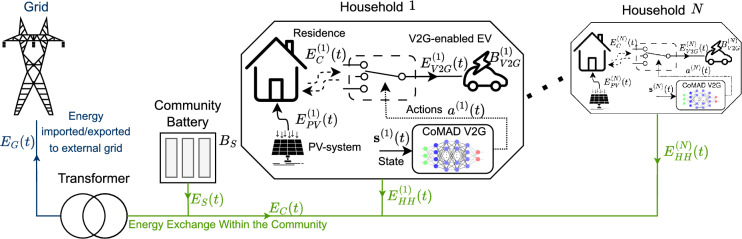
System model illustration showing energy flows in the community and the placement of the proposed CoMAD V2G solution.

A community comprising *N* households, as illustrated in Fig. 1, is considered. Each household is equipped with a PV system and a single V2G vehicle. The community also features a shared ESS with a capacity of $B_{S}$, capable of storing energy collected by the community. It is further assumed that the community can exchange energy with the DSO. Each element within the community such as residential base consumption, the shared ESS, and V2G vehicles, affects energy exchange in distinct ways, which are detailed in this section. For additional clarity, the notations employed throughout this paper are listed in Table 1.

**Table 1:** Notation used in the system model.

Notation	Description	Notation	Description
*N*	Number of households	*n*	Household index
*t*	Time step	$\tau$	Time step size
$B_{S}$	ESS capacity	$B_{V2G}^{(n)}$	*n*-th household V2G capacity
$P_{PV}^{(n)}$	Installed PV system power	$\eta _{PV}$	PV efficiency
$E_{CONS}^{(n)}(t)$	*n*-th residence base load energy consumption	$E_{PV}^{(n)}(t)$	*n*-th household generated PV energy
$E^{(n)}_{V2G}(t)$	*n*-th household V2G energy exchange	$e^{(n)}_{V2G}(t)$	*n*-th household energy available in the V2G element
$P_{V2G}^{(n)}$	V2G element charging/discharging power	$\eta _{V2G}$	V2G element charging/discharging efficiency
$E_{HH}^{(n)}(t)$	*n*-th household consumption	$E_{C}(t)$	Energy consumption of the Community
$E_{S}(t)$	Energy exchange between ESS and community	$e_{S}(t)$	Energy available in the ESS
$P_{S}$	ESS charging/discharging power	$\eta _{S}$	ESS charging/discharging efficiency
$\Delta _{S}(t)$	Energy ESS exchanges in time step *t*	*M*	Number of ESS charging cycles
$p_{S}(t)$	Sell price of energy to grid in timestep *t*	$p_{B}(t)$	Buy cost of energy from grid in timestep *t*
$\lambda _{C}(t)$	Cost of energy in time step *t*	$\Lambda _{C}$	Total cost of energy per household
*T*	Number of timesteps	$E_{G}(t)$	Energy imported/exported to external grid

### Energy consumption of the community

The energy consumption, i.e., residential base consumption, of the *n*-th household, $n \in {1,2,\dots ,N}$, at time step *t* is denoted as $E_{CONS}^{(n)}(t)$. This energy load is primarily determined by residents’ daily routines and preferences, as well as the automated operation of systems such as Heating, Ventilation and Air Conditioning (HVAC), which respond to either pre-set preferences or environmental conditions. In the considered system, this load is assumed to be fixed and not subject to external control, thereby ensuring the preservation of user comfort at all times. User comfort is defined as the fulfillment of occupants’ physiological and psychological needs related to indoor environmental quality such as thermal, visual, acoustic, and air quality^25^. To uphold this standard, the proposed solution deliberately refrains from controlling any loads that could impact these needs, such as direct HVAC management. This assumption is considered essential, as modifying household energy loads without explicit user involvement could disrupt the intended automation and compromise comfort. Consequently, the approach does not include direct manipulation of household energy consumption.

Each household is also equipped with a PV system that generates renewable energy, denoted as $E_{PV}^{(n)}(t)$. The energy generated by the PV system is primarily influenced by the installed capacity, denoted as $P_{PV}^{(n)}$, efficiency of PV energy conversion $\eta _{PV}$, and the amount of solar radiation received, which varies throughout the day and across seasons. This variation introduces dynamic factors that affect both the availability of renewable energy and the degree to which households can be self-sufficient at different times. For example, during sunny summer months, PV generation may exceed household consumption, potentially leading to an energy surplus, while in winter, energy generation may be significantly lower, increasing reliance on external energy sources, i.e., energy from the DSO.

The V2G-enabled EV adds to the dynamic energy exchange within each household, as the V2G is not always connected due to the inhabitants of the household using it for commuting, travel, grocery shopping, etc. The V2G energy exchange, as denoted with $E^{(n)}_{V2G}(t)$, is proportional to charging or discharged power $P_{V2G}^{(n)}$, i.e., $E^{(n)}_{V2G}(t)= P_{V2G}^{(n)} \tau$, where $\tau$ is time step size. Furthermore, the V2G battery capacity is denoted as $B_{V2G}^{(n)}$, available energy in the EV with $e_{V2G}^{(n)}(t)$, i.e., $e_{V2G}^{(n)}(t) \in [0, B_{V2G}^{(n)}]$, and charging/discharging efficiency with $\eta _{V2G}$, where $\eta _{V2G} \le 1$. Often, the energy generated by the PV system cannot be stored in the V2G due to the V2G not being present, its battery already being at full capacity, or simply due to limits on the charging power.

The combined energy consumption of the household at time step *t* is then:

1 $$\begin{aligned} E_{HH}^{(n)}(t) = E_{PV}^{(n)}(t) - E_{CONS}^{(n)}(t) - E^{(n)}_{V2G}(t). \end{aligned}$$

The net energy from the household may be either positive or negative, where a negative total indicates that the household is drawing energy from the community, and a positive total signifies that the household is supplying energy back to the community.

Finally, the total energy consumption of the community comprising *N* households is defined as follows:

2 $$\begin{aligned} E_{C}(t) = \sum _{n=1}^{N} E_{HH}^{(n)}, \end{aligned}$$

where a negative total consumption indicates that energy needs to be supplemented by the ESS or by the grid if the ESS is empty, while a positive total indicates that excess energy can be stored in the battery or transferred to the grid if the ESS is at full capacity.

### Energy exchange in the community ESS

The shared ESS allows *N* households to collectively store their surplus energy and draw from it whenever needed. This ESS has a finite storage capacity, denoted as $B_{S}$, which restricts the total amount of energy it can hold. The energy available in the shared ESS is represented by $e_{S}(t)$, where $e_{S}(t) \in [0, B_{S}]$. Additionally, the system is limited by a maximum charging power, $P_{S}$, which restricts the amount of energy that can be exchanged with the community in each time step. This power limitation ensures that the ESS operates within safe and practical charging and discharging rates, supporting effective energy management across the community. Consequently, the amount of energy the shared ESS can exchange, denoted by $\Delta _{S}(t)$, is limited and depends on whether the system requires energy from the ESS or is storing the energy, as well as the current state of charge of the ESS. This can be expressed as follows:

3 $$\begin{aligned} \Delta _{S}(t) = {\left\{ \begin{array}{ll} P_{S} \tau \phantom {AAAAAAAA} \text { if } \phantom {A} E_{C}(t) \ge 0 \text { and } B_{S} - e_{S}(t-1) \eta _{S} \ge P_{S} \tau \\ B_{S} - e_{S}(t-1) \phantom {.A} \text { if } \phantom {A} E_{C}(t) \ge 0 \text { and } B_{S} - e_{S}(t-1) \eta _{S}< P_{S} \tau \\ - P_{S} \tau \phantom {AAAAAAA} \text { if } \phantom {A} E_{C}(t)< 0 \text { and } e_{S}(t-1) \ge P_{S} \tau \\ - e_{S}(t-1) \phantom {AAA.} \text { if } \phantom {A} E_{C}(t)< 0 \text { and } e_{S}(t-1) < P_{S} \tau \end{array}\right. }. \end{aligned}$$

Note that in the equation above $\eta _{S}$ represents charging and discharging efficiency of ESS. The first case represents a situation when the community generates excess energy, and the shared ESS is not yet fully charged. The second case represents a situation when community energy can be stored, but the ESS is almost full, hence only a portion of the possible energy that could be stored in time step, *t*, is actually stored. In contrast, the last two cases represent situations when the community uses the stored energy, with the final case describing a situation in which the ESS is nearly empty, allowing only a fraction of the required energy to be taken. Therefore the change in ESS available energy is as follows:

4 $$\begin{aligned} e_{S}(t) = {\left\{ \begin{array}{ll} e_{S}(t-1) + \eta _{S} \Delta _{S}(t) \phantom {AA} \text { if } \phantom {A} E_{C}(t) \ge 0 \\ e_{S}(t-1) + \Delta _{S}(t) \phantom {AAAA} \text { if } \phantom {A} E_{C}(t) < 0 \end{array}\right. }. \end{aligned}$$

The total energy exchanged between the ESS and the community at each time step *t* can then be expressed as:

5 $$\begin{aligned} E_{S}(t) = {\left\{ \begin{array}{ll} \min \bigg (E_{C}(t), \Delta _{S}(t) \bigg ) \phantom {AAAA} \text { if } \phantom {A} E_{C}(t) \ge 0 \\ \eta _{S} \max \bigg (E_{C}(t), \Delta _{S}(t) \bigg ) \phantom {AA} \text { if } \phantom {A} E_{C}(t) < 0 \end{array}\right. }. \end{aligned}$$

The energy imported to and exported from the community to the external grid, $E_{G}(t)$, is calculated as follows:

6 $$\begin{aligned} E_{G}(t) = {\left\{ \begin{array}{ll} \max \bigg (E_{C}(t) -\Delta _{S}(t), 0 \bigg ) \qquad \text { if } \phantom {A} E_{C}(t) \ge 0 \\ \min \bigg (E_{C}(t) - \Delta _{S}(t), 0 \bigg ) \qquad \text { if } \phantom {A} E_{C}(t) < 0 \end{array}\right. }. \end{aligned}$$

Lastly, a metric *M* is defined to quantify the number of charging cycles over a period *T*. A single charging cycle is considered complete when the total charge added across multiple charging sessions equals 100% of the shared ESS capacity. Accordingly, the number of charging cycles is defined as follows:

7 $$\begin{aligned} M = \frac{1}{B_{S}} \sum _{t=1}^T E_{S}(t). \end{aligned}$$

With the energy dynamics within the community established, the next step is to determine the community’s energy cost.

### Cost of energy in a shared community

The smart community, as a collective entity, trades energy directly on the open energy market^26^. This implies that the community pays the prevailing market prices for energy as a whole while also acting as a seller of excess energy. Note that the prices paid by the community differ from the rates individual households typically pay for electricity. This highlights another advantage such a system can offer by acting as a unified entity. The monetary benefits of being part of such a community are distributed accordingly. Consequently, the cost of energy exchanged with external grids is determined as:

8 $$\begin{aligned} \lambda _{C}(t) = {\left\{ \begin{array}{ll} p_{S}(t) E_{G}(t) \qquad \text { if } \phantom {A} E_{G}(t) \ge 0 \\ p_{B}(t) E_{G}(t) \qquad \text { if } \phantom {A} E_{G}(t) < 0 \end{array}\right. }. \end{aligned}$$

where $p_{S}(t)$ represents the selling price, and $p_{B}$ represents the buying price. Positive energy exchange with the grid $E_{G}(t)$ indicates energy is exported to the grid, while negative $E_{G}(t)$ represents energy imported from the grid. Therefore, it is possible to determine the average cost of one household energy exchange over a period *T* as follows:

9 $$\begin{aligned} \Lambda _{C}= \frac{1}{N} \sum _{t=1}^{T} \lambda _{C}(t). \end{aligned}$$

### Challenges of non-intrusive energy optimization in smart communities

In the considered community, energy dynamics are influenced by variable household consumption, fluctuating electricity prices, and the availability of V2G-enabled EVs. Household energy usage varies across daily, weekly, and seasonal cycles, while EVs depend on user behavior, affecting their availability for energy storage and discharge. Additionally, dynamic electricity pricing introduces uncertainty but also offers opportunities for cost optimization. However, improving energy allocation within the community should not come at the cost of user comfort or require changes in their behavior. In other words, a solution should be non-intrusive.

The most significant energy consumption element in the system is the V2G-enabled EVs, and controlling its charging and discharging patterns is key to optimization. At the same time, a shared ESS can serve as temporary storage for excess energy from households when EVs are unavailable or when PV systems are not generating energy, such as during nighttime. To that end, the proposed solution focuses on optimizing the charging and discharging patterns of V2G-enabled EVs. This approach ensures that the solution is non-intrusive, as it does not alter user behavior patterns. Additionally, the proposed solution maintains a minimum required charge level for the V2G-enabled EV, ensuring that the vehicle remains available for use at all times. This guarantees that user comfort is not affected and prevents the vehicle from running out of energy when needed.

To address the dynamic nature of the system and develop a solution capable of adapting to individual consumption patterns, external factors like seasonal changes, and real-time pricing variations. For such a system, DRL was identified as the most effective approach. Furthermore, DRL allows for non-intrusive optimization by enabling seamless control of V2G charging and discharging, ensuring user behavior remains unchanged while reducing costs and enhancing energy management in the community.

## CoMAD V2G solution

The proposed solution adopts a distributed MARL^27^ approach, where each agent controls the charging of the V2G-enabled EV at its respective household in a non-intrusive manner. The MARL framework enables the design of a scalable solution for highly complex environments, which is essential given the dynamic nature of V2G-enabled EVs entering and leaving the community. Furthermore, employing MARL ensures robustness, as suboptimal behavior by one agent at a particular household can be compensated by other agents in the community. This leads to a system capable of better overall performance and resilience. Additionally, decisions are made in a decentralized manner, preserving the privacy of individual households. Only local information from each household and relevant shared information are utilized, ensuring efficient and privacy-aware operation.

**Table 2:** Notation used in the MARL.

Notation	Description	Notation	Description
${\textbf {s}}^{(n)}(t)$	State	*w*	State window size
$a^{(n)}(t)$	Action	$r^{(n)}(t)$	Reward
$e_{rMAX}$	Maximal reward for V2G charge level	$e_{rMIN}$	Minimal reward for V2G charge level
$p_{rMAX}$	Maximal reward cost price normalization	$p_{rMIN}$	Minimal reward cost price normalization
$E_{rCONS}$	Consumption reward normalization	$\alpha _{V2G}$	V2G energy reward weight
$\alpha _{S}$	Energy exchnage reward weight	$\alpha _C$	Energy trading reward weight
$Q^{(n)}(t)$	Policy network	$Q^{'(n)}(t)$	Target network
$\theta ^{(n)}(t)$	Weights of policy network	$\theta ^{'(n)}(t)$	Weights of target network
$\mathscr {D}^{(n)}$	Replay memory	*D*	Replay memory size
*J*	Batch size	$\zeta$	Soft update value of target network

### State, actions, and reward

In this subsection, the state, actions, and reward signal for the proposed CoMAD V2G solution are defined, and the DRL-related notations are listed in Table 2.

The **state** space employed consists of the information available to each agent for its respective household, together with global information accessible to the entire community. Individual information regarding the performance or energy consumption of each household is not made available to the agent, thereby ensuring that confidential information is not shared between households. The selected state relies on seven variables, as follows:

10 $$\begin{aligned} {\textbf {s}}^{(n)}(t)&= \{E_{PV}^{(n)}(t-w),..., E_{PV}^{(n)}(t),E^{(n)}_{V2G}(t-w),...,E^{(n)}_{V2G}(t), e^{(n)}_{V2G}(t-w),...,e^{(n)}_{V2G}(t), e_{S}(t-w),..., e_{S}(t), \nonumber \\&\Delta _{S}(t-w),...,\Delta _{S}(t),p_{S}(t-w),...p_{S}(t),p_{B}(t-2),...p_{B}(t)\}. \end{aligned}$$

Note that *w* is the window size of the time series for each variable used in the state space. The state space consists of information from the *n*-th household regarding the household-generated PV energy $E^{(n)}_{PV}$, the V2G energy exchange between the household and V2G enabled EV, i.e., $E^{(n)}_{V2G}$, and the energy available in the EV, i.e., $e^{(n)}_{V2G}$. From the community, each agent receives information regarding the energy available in the shared ESS $e_{S}(t)$, the energy exchange between the ESS and the community $\Delta _S(t)$, as well as the sell price $p_S(t)$ and buy price $p_B(t)$ of energy. Combined, the state ensures that the agent has information about key performance indicators of the household for which it is making decisions, the state of the ESS, and, through the energy exchange between the ESS, the community, and the external grid, how well other households perform and interact. Furthermore, the buy and sell prices reflect the global conditions of the grid. For example, during the summer, when there is plenty of renewable energy available in the system, prices are low, while in the winter, prices tend to be higher. Such information can aid the agent in making more informed decisions about which energy sources to employ.

To ensure a non-intrusive solution, the agent’s sole decision is limited to determining the charging power for the V2G-enabled EV, provided it is connected to the household. Accordingly, the actions are restricted to the following five options:

11 $$\begin{aligned} a^{(n)}(t) = {\left\{ \begin{array}{ll} P_{V2G}^{(n)}, \phantom {AAA} \text {(charging with full power)} \\ \frac{1}{2}P_{V2G}^{(n)}, \phantom {AA} \text {(charging with half of power)} \\ 0, \phantom {AAAAA.}\text {(no-change)} \\ -\frac{1}{2}P_{V2G}^{(n)}, \phantom {A} \text {(discharging with half of power)} \\ -P_{V2G}^{(n)}, \phantom {AA} \text {(discharging with full power)} \\ \end{array}\right. }. \end{aligned}$$

This means that the energy in the element V2G is updated in the time step *t* as:

12 $$\begin{aligned} e_{V2G}^{(n)}(t) = e_{V2G}^{(n)}(t-1) + a^{(n)}(t) \eta _{V2G} \tau . \end{aligned}$$

Consequently, the agent’s action affects only the amount of energy available within the system. A precaution is implemented by specifying a minimum energy threshold that the V2G-enabled EV must retain in order to permit discharging. In this study, this threshold is set at $30\%$, while in practical deployment, it could be adjusted based on user habits and preferences (incl. planned longer trips) via an appropriate application. This precaution prevents complete battery discharge and ensures that a sufficient amount of energy remains available to the user of the V2G-enabled EV.

The reward each agent receives is designed to incentivize the agent to achieve three objectives. The first objective is to charge the V2G-enabled EV such that its energy remains within a certain predefined limit. The second objective is to ensure that the energy the community as a whole takes from the grid is kept to a minimum. Lastly, the third objective is to maximize the possible gains from energy trading that the community might receive. In other words, the goal is to minimize the amount of money households pay for electricity. To achieve the first objective, the V2G charging reward is defined as:

13 $$\begin{aligned} r_{V2G}^{(n)}(t)= {\left\{ \begin{array}{ll} 1+\frac{ e_{V2G}^{(n)}(t) / B_{V2G}^{(n)}}{e_{rMAX}-e_{rMIN}}, \phantom {AA} \text {if } e_{rMIN} \le \frac{e_{V2G}^{(n)}(t)}{B_{V2G}^{(n)}} \le e_{rMAX}\\ \frac{e_{V2G}^{(n)}(t)}{B_{V2G}^{(n)}} -1, \phantom {AAA.} \text {otherwise} \\ \end{array}\right. }, \end{aligned}$$

where $e_{rMAX}$ and $e_{rMIN}$ represent the maximum and minimum levels of charge in the V2G-enabled EV acceptable in the community. Note that the above reward function incentivizes the agent to charge the EV when the amount of energy is lower than the set minimum level and to discharge it when the level is above the set maximum value. The reward function that disincentives the energy exchange between the community and the external grid is defined as follows:

14 $$\begin{aligned} r_{S}^{(n)}(t)= {\left\{ \begin{array}{ll} \frac{E_{S}(t)}{E_{rCONS}}, \text {if } -1 \le \frac{E_{S}(t)}{E_{rCONS}} < 0\\ 0, \phantom {AAA.} \text {if } \frac{E_{S}(t)}{E_{rCONS}} \ge 0 \\ -1 , \phantom {AA} \text {if } -1 > \frac{E_{S}(t)}{E_{rCONS}} \\ \end{array}\right. } \end{aligned}$$

where $E_{rCONS}$ is a constant used to normalize the consumption reward. Limiting the consumption reward to a predefined interval provides stability. For example, very high consumption could result in an extremely high negative reward, prompting agents to avoid such states at all costs, which could, in turn, impact the consumption of the entire community. Lastly, the objective to minimize the cost has similar limitations in place, as follows:

15 $$\begin{aligned} r_{C}^{(n)}(t)= {\left\{ \begin{array}{ll} \frac{\lambda _{C}(t)}{p_{rMAX}}, \phantom {AA.} \text {if } 0 \le \lambda _{C}(t) \le p_{rMAX} \\ 1, \phantom {AAAAA.} \text {if } \lambda _{C}(t)> p_{rMAX} \le 0 \\ - \frac{\lambda _{C}(t)}{p_{rMIN}}, \phantom {A.} \text {if } p_{rMIN} \le \lambda _{C}(t) \le 0 \\ -1, \phantom {AA} \phantom {AA} \text {if } p_{rMIN} >\lambda _{C}(t) \\ \end{array}\right. } \end{aligned}$$

where $p_{rMAX}$ and $p_{rMIN}$ are the maximum and minimum values for reward function normalization, respectively. By limiting the maximum value of the reward function, it is possible to reduce the impact of outlier states that are typically only experienced during the training of the agents, thus also allowing for faster training of the agent.

With individual rewards assigned to each targeted objective for the agents, the overall reward function is defined as follows:

16 $$\begin{aligned} r^{(n)}(t)= \underbrace{ \alpha _{V2G} r_{V2G}^{(n)}(t) }_{\text {Available V2G energy reward}} + \underbrace{\alpha _{S} r_{S}^{(n)}(t) }_{\text {Energy exchange reward}} + \underbrace{ \alpha _{C} r_{C}^{(n)}(t) }_{\text {Energy trading reward}}. \end{aligned}$$

$\alpha _{V2G}$, $\alpha _{S}$, and $\alpha _C$ represent the weights assigned to each of the objectives, i.e., the individual rewards. These weights are crucial for the balance of the final reward that each agent receives for each decision, since the rewards for energy exchange ($r_{S}^{(n)}(t)$) and cost ($r_{C}^{(n)}(t)$) are often zero, while the reward for the available energy in the V2G-enabled EV($r_{V2G}^{(n)}(t)$) is never zero. As a result, the factor $\alpha$ for the objective of charging V2G should be lower than for the other two goals to ensure a balance between the rewards.

### CoMAD V2G algorithm

Algorithm 1 outlines the proposed solution for charging and discharging V2G-enabled EVs in a community with a shared ESS. The solution is based on the well-known Deep Q-Network (DQN) algorithm with prioritized experience replay^28^ and is adapted to the specific scenario under consideration. First, the policy and target networks for *N* agents are initialized. These weights are initialized randomly during the training phase, whereas in the deployment phase, pre-trained networks are loaded. In line 2, the initial state is observed, and charging and discharging decisions are made in parallel for every household.

**Algorithm 1:**
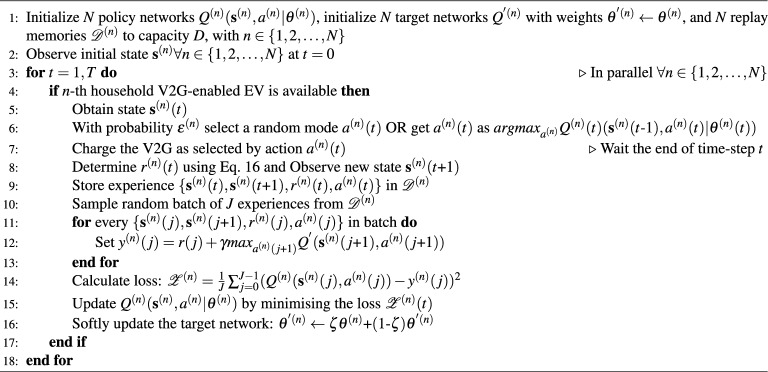
Proposed community multi-agent deep reinforcement learning vehicle-to-grid(CoMAD V2G) algorithm

At each step, the decision made by the *n*-th agent for the *n*-th household is only executed if the V2G-enabled EV is connected (line 4). The process begins with the agent receiving the state space for the time step *t* (line 5), followed by the selection of an action (line 6). CoMAD V2G employs an $\epsilon$-greedy approach for action selection, which means that the action is selected randomly with a probability of $\epsilon$. During training, $\epsilon$ starts with a high value and gradually decreases over time until it reaches a predefined minimum value. As soon as an action is selected, the element V2G is charged or discharged according to the action for the duration of the time step *t* (line 7). At the end of the time step, the agent observes a new state, $\textbf{s}^{n}(t)$, and calculates the reward using Eq. 16. The agent then stores the experience, which is represented as a tuple of the current state, the next state, the reward and the action (i.e. ${ {\textbf {s}}^{(n)}(t), {\textbf {s}}^{(n)}(t\text {+}1), r^{(n)}(t), a^{(n)}(t) }$), in the replay memory (line 9). The neural networks are updated in several steps as follows: First, *J* experiences are sampled from the replay memory (line 10), then the expected value for each experience is calculated (line 12) and the loss is calculated (line 14). The Q values are then updated (line 15), and finally a soft update of the target network is performed (line 16).

### Hyperparameters

Each agent uses a policy and target neural network with four hidden layers. The input layer consists of the same number of neurons as the number of different variables in the state space, i.e., 7*w*. The first hidden layer consists of 128 neurons, the second and third layers consist of 256 neurons each, and the last hidden layer consists of 128 neurons, the same as the first hidden layer. The output layer consists of 5 neurons, each representing one of the available actions at the agent’s disposal. The activation function at each layer is the standard ReLU function. The rest of the hyperparameters, which were obtained using exhaustive search, are listed in Table 3.

**Table 3:** CoMAD V2G solution parameters.

Hyperparameter	Value	Hyperparameter	Value	Hyperparameter	Value
$\alpha _{V2G}$	1	$\alpha _{S}$	10	$\alpha _C$	10
$e_{rMAX}$	0.8	$e_{rMIN}$	0.2	$E_{rCONS}$,	1.0 kWh
$p_{rMAX}$	$0.2 \frac{\text {{\EUR \text{\euro } }}}{kWh}$	$p_{rMIN}$	$-0.5 \frac{\text {{\EUR \text{\euro } }}}{kWh}$	*w*	96
*D*	$10^5$	*J*	512	$\zeta$	$10^{-3}$
Start epsilon value $\epsilon ^{(n)}$	0.99	Minimal epsilon value $\epsilon ^{(n)}$	0.25	Epsilon decay	0.9998
Loss function	MSE	Optimizer	Adam		

Table 3 lists the parameter values specific to the proposed solution, including variables used for reward function normalization. These values were determined by analyzing the training data and ensuring minimal impact from outliers in consumption or electricity cost on the reward outcome. Additional parameters, such as $\alpha _{V2G}$, $\alpha _{S}$, and $\alpha _C$, were selected based on performance optimization during agent training.

## Validation

This section validates the proposed CoMAD V2G solution^29^ using multiple real-world datasets to model the community under consideration with a shared ESS.

### Simulation setup, training, and baseline descriptions

To design a realistic simulation for the community under consideration, consisting of *N* households as depicted in Fig. 1, several data sources were utilized. The location was determined by selecting the authors’ country and situating the community in a sub-urban area, thereby enabling the use of relevant real-world data. Various tools and sources were then employed to achieve a realistic system model. Specifically, real-world data collected between 2022 and 2023 were used to model the following components:

- **Household consumption**: Household consumption, i.e., $E_{CONS}(t)$, was modeled for two years using LoadProfileGenerator^6^. These consumption traces represent realistic behavior, with the number of inhabitants varying randomly between 2 and 5 per household.
- **PV system:** To capture the different aspects and effects of weather dynamics on electricity generation, real data is used to model the electricity generation of a PV system over time. It is assumed that each household has a PV system with an area of $30m^2$ and an efficiency $\eta _{PV}$ as listed in Table 4. The amount of energy generated is proportional to the direct radiation (in $W/m^2$) obtained from the Open Meteo website^7^ using the location at N $46^\circ$ $4'$ $59.88'$, E $15^\circ$ $0'$ $0''$. This location in Slovenia was selected by the authors as it represents a region where such a community would be feasible.
- **V2G modeling**: The presence of EVs, their energy consumption, i.e., $e_{V2G}(t)$ at the time the vehicles returned to the household, and their capacity, i.e., $B_{V2G}$, is modeled using the tool *emobpy*^8^. The resulting models of EV battery consumption account for driving energy, accessory loads, e.g., heating and cooling, and charging and discharging losses, including self-discharge losses.
- **Electricity pricing**: The cost of buying or selling electricity, i.e., $p_{s}$ and $p_{B}$, as paid by the community, was obtained from the BSP Southpool website^9^. The pricing reflects the selected region of the simulated community.

**Table 4:** Simulation parameters.

Parameter	Value	Parameter	Value	Parameter	Value
$P_{S}$	20 kWh	$\eta _{s}$	0.95	*T*	35040
$P_{V2G}$	7 kWh	$\eta _{V2G}$	0.9/0.95 (charge/discharge)	$\tau$	15 min
PV surface size	30 m^2^	$\eta _{PV}$	0.275		

The evaluation also includes a comparison of the proposed solution against alternative modes of charging and discharging of V2G, namely:

- **Immediate mode**: In this mode, the V2G-enabled EV is charged as soon as it connects to the community grid. This mode focuses on convenience and ensures that the vehicle battery is charged as quickly as possible without taking into account factors such as electricity prices or conditions. While this mode is simple and ensures the availability of energy for the user, it may not optimize charging costs or the balance of energy use in the community.
- **Night mode**: In this mode, the V2G-enabled EV charging is limited to nighttime hours when electricity prices are generally lower due to lower demand on the grid. By charging only at night, in the considered system this is between 11pm and 8am. Such a mode aims to reduce electricity costs for households while indirectly supporting grid stability.
- **Individual DLR mode**: This mode represents a more intelligent and dynamic charging approach. Each agent operates independently but has the same state information and available actions as defined in the proposed framework. In contrast to the simpler modes, the individual DRL mode uses a decision-making process based on the agent’s understanding of its environment. It optimizes decisions individually rather than collaboratively, relying on local conditions within the household, such as energy consumption, EV status, and electricity prices. The goal is to optimize charging and discharging decisions to balance multiple objectives such as minimizing costs, maintaining grid stability and ensuring sufficient energy availability.

The proposed CoMAD V2G solution and the individual DRL mode were trained on datasets from 2022, while the validation results presented in the next subsections are based on data from 2023.

### Quarterly analysis of energy exchange dynamics

**Fig. 2:**
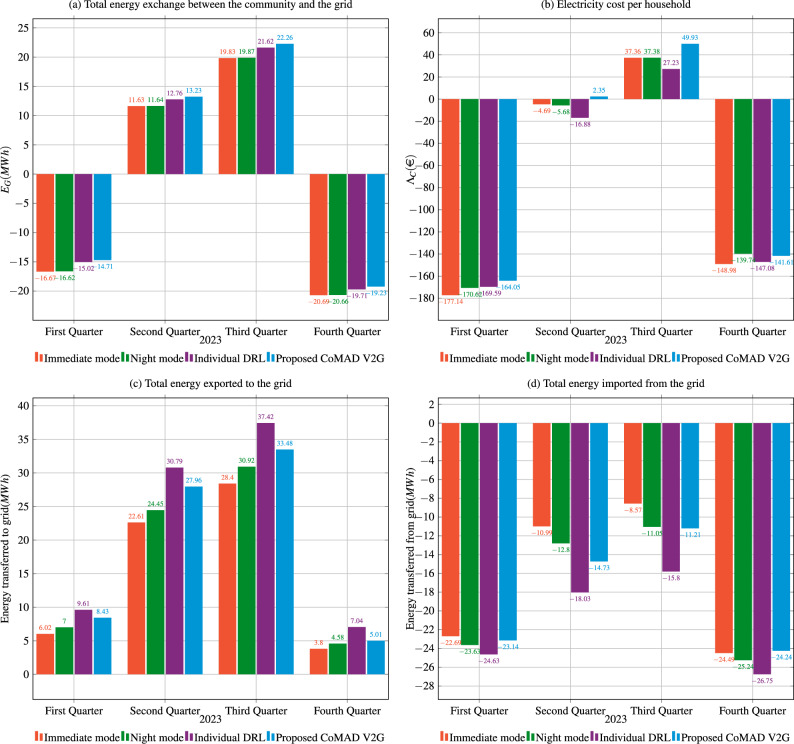
2023 quarterly analysis of total energy exchange between the community and the grid, electricity costs, energy exported to the grid, and energy imported from the grid. $N=20$ and $B_S=200kWh$.

Figure 2a shows the total energy exchange, with positive values representing energy exported to the grid and negative values representing energy imported from the grid. As expected, in the first quarter of 2023 due to the dependence on PV, the community has to import a significant amount of energy to compensate lack of energy generation during winter months. In contrast, in the second and third quarters, the community exports energy to the grid, while in the last quarter of 2023, when the generation of PV decreases, it imports energy again. In each quarter, the proposed CoMAD V2G solution outperforms the other approaches in terms of energy exchange with the grid. For example, in the first and last quarters, the negative exchange indicates that it imports the least amount of energy from the grid, while in the second and third quarters, it exports the most in comparison to other approaches. This indicates that strategically deciding when to charge and discharge the V2G system critically impacts the overall energy exchange between the community and the rest of the grid, with the shared ESS serving as a buffer to store and compensate when necessary

Figure 2c shows the amount of energy exported by each solution per quarter, while Fig. 2d shows the energy imported by each solution. The proposed solution exports and imports less energy compared to the predefined operating modes (immediate and night mode), while the individual DRL solution exchanges significantly more energy. This behavior probably results from the fact that the individual strategy acts rather reactively and without a holistic understanding of the community’s performance. Consequently, this approach leads to higher energy costs, as shown in Fig. 2b.

Figure 2b shows the costs of electricity for each household in each quarter. Positive values represent income, while negative values represent payments for electricity. The proposed solution performs better than all other approaches in each quarter. In contrast, the individual DRL solution leads to the highest costs for households in almost every quarter. Surprisingly, the cost difference between charging EV in the immediate mode and charging during the night (night mode) is smaller than expected, amounting to only about 20€ over the course of a year. The individual DRL solution is the most expensive, which is due to the fact that it exchanges the largest amount of energy (both imports and exports) among the four approaches investigated.

### Impact of community size on energy exchange and charging cycles

**Fig. 3:**
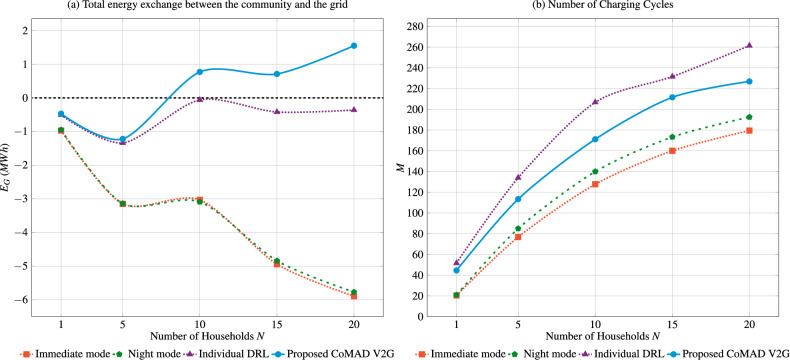
Total energy exchange between the community and the grid, and the number of charging cycles, shown as a function of the number of households. $B_S=200\,\text{kWh}$.

The second experiment analyzes the impact of community size on the total energy exchanged with the grid and the number of charging cycles, as illustrated in Fig. 3. Notable trends are observed as the number of households increases.

In particular, the individual DRL and the proposed CoMAD V2G solutions exhibit similar performance when the community is small, with almost identical results when the community consists of only one household. The proposed solution slightly outperforms the individual DRL solution in a community of five households, as shown in Fig. 3a. Additionally, the results indicate that the benefits of intelligent solutions like CoMAD V2G increase with the number of households in the community, as the capacity of the shared ESS remains the same. Specifically, the total amount of energy exchanged with the grid decreases and even becomes positive, meaning that the community delivers more energy to the grid than it consumes. In contrast, for conventional approaches (immediate and night modes), the benefits of a larger community diminish as the number of households increases.

The larger the community, the more charging cycles the ESS performs annually, as shown in Fig. 3b. Interestingly, the individual DRL solution results in the highest number of charging cycles, followed by the proposed CoMAD V2G solution, the night mode, and finally the immediate mode, which requires the fewest ESS charging cycles. Perhaps most notable is the observed difference in energy exchange. For instance, when the community consists of 20 households, the difference between the proposed CoMAD V2G solution and the immediate mode in terms of total energy exchange is approximately 7.6 *MWh* annually. In such a case, the proposed solution enhances the energy autonomy of the community by substantially lowering grid energy imports, up to 350 kWh per household annually compared to heuristic approaches, and approximately 100 kWh compared to an alternative MARL-based method. Notably, these results are achieved with only around 40 additional charging cycles per year.

### Impact of shared ESS size on energy exchange and charging cycles

**Fig. 4:**
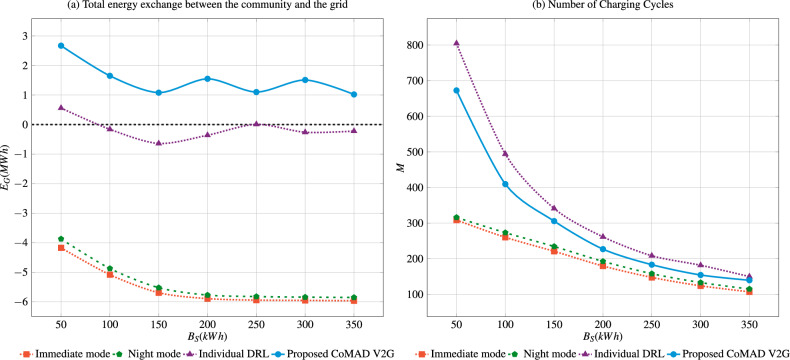
Total energy exchange between the community and the grid, and the number of charging cycles, shown as a function of the Shared ESS size. $N=20$.

Figure 4a illustrates how the size of the shared ESS impacts the total energy exchange and the number of charging cycles. The observed behavior is intriguing, as the performance of each approach generally improves with a smaller ESS size. This result is primarily influenced by the limitation of the charging and discharging power. Interestingly, the performance of each DRL approach remains relatively constant regardless of the ESS size. It is important to note that the discharge power of the battery ($P_{S}$) has a significant impact on the results. In this analysis, the $P_{S}$ is kept constant regardless of the shared ESS size, whereas in real-world scenarios the charging and discharging power often scales with the size of the battery.

On the other hand, the size of the shared ESS has a much greater impact on the number of charging cycles, as shown in Fig. 4b. The larger the ESS, the fewer charging and discharging cycles are required. Doubling the ESS capacity, for example, almost halves the number of cycles for both the individual DRL solution and the proposed CoMAD V2G solution. While the reduction can also be observed for the conventional approaches (immediate mode and night mode), it is less pronounced. Interestingly, the individual DRL solution leads to a higher number of charging and discharging cycles compared to the proposed CoMAD V2G solution, although the total energy exchange between the community and the grid is larger in the proposed solution. This highlights the efficiency of the CoMAD V2G strategy in managing energy exchange and battery utilization.

### Cost analysis

**Fig. 5:**
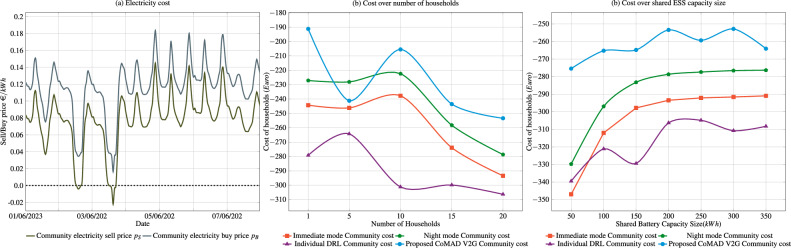
The left graph presents the variation in electricity cost during the first week of June 2023. The middle graph displays the change in cost per household as a function of the number of households ($B_{S}=200,\textrm{kWh}$). The rightmost graph illustrates the relationship between electricity cost and ESS capacity ($N=20$).

In the final experiment, shown in Fig. 5, evaluates the proposed solution in terms of the electricity costs incurred per household. Fig. 5a illustrates how the community’s electricity cost function varies over time between 1st of June 2023 and 7th of June 2023. On certain days in the selected period, the costs even reach negative values, indicating that the community receives payments for providing electricity to the grid. Note that the difference between sell ($p_{S}$) and buy price ($p_{B}$) is 0.03858€/*kWh*, a network distribution fee. In this work, Slovenian national average network distribution price for 2022 and 2023 is used to model the buy price.

Figure 5b shows how the annual electricity costs per household change with the number of households in the community. Interestingly, the general trend for each approach shows that the cost per household increases with increasing community size, suggesting that being part of a larger community becomes less cost-effective. Nevertheless, the proposed solution shows the best performance as it has the lowest annual electricity costs. The increase in cost per household with more households can be attributed to the fact that the shared ESS capacity is divided among a larger number of households, resulting in a higher cost per household.

Figure 5c illustrates how the cost for a community consisting of 20 households changes as the size of the shared ESS increases. The trend shows that as the capacity of the shared ESS increases, the cost per household decreases, a pattern that is clearly visible for both the immediate and night modes. The proposed CoMAD V2G solution demonstrates superior performance, resulting in lower costs per household for every ESS size. It is important to note that both the proposed solution and the individual DRL solution exhibit variations in performance. These oscillations are a result of the dynamic and adaptive nature of DRL agents, as they explore and exploit different sets of actions. In addition, the proposed approach reduces household electricity costs by up to 25% in comparison to baseline methods.

### Discussion

Results show that the proposed CoMAD V2G solution increases the energy autonomy of a PED, defined as a district that produces at least as much energy as it consumes on an annual basis. As shown in Figs. 3 and 4, the intelligent management provided by CoMAD V2G enables the community to export more energy to the grid than it imports, a result not achieved with other control methods. It is important to note that the considered community generates sufficient energy to be classified as a PED; however, only with the integration of the shared ESS and the proposed solution it becomes significantly more energy autonomous. This highlights the importance of employing advanced intelligence for dynamic energy management, as it not only ensures community sustainability but also optimizes usage of resources and minimizes costs.

In terms of electricity costs, analysis, shown in Fig. 5, shows that the timing of charging and discharging the V2G system has a significant impact on energy savings and increases the benefits of sharing an ESS. However, the previous analysis does not fully account for the cost savings that result from sharing ESS. For example, if the community operates under an immediate V2G charging policy, the average electricity cost per household ($N=20$, $B_S=200$ kWh) would be approximately 866 €. In contrast, the proposed solution reduces these costs to 253 €, resulting in annual savings of over 600 € per household. However, a more comprehensive assessment must also take into account the initial investment. Assuming battery cost of 500 €/kWh, a shared 200 kWh ESS would require an initial investment of around 100*k* €. In a community of $N=10$ households with a shared storage this results in the intial cost of 10*k* € per household, while in a larger community, e.g., $N=20$, the initial cost would be reduced to 5*k* € per household. Despite these initial costs, the financial benefits of participating in such a shared storage system are significant. In addition, larger energy communities provide households access to the electricity market, a privilege typically unavailable to an individual household. This market participation, combined with optimized storage usage, further improves the cost efficiency and practicality of forming larger energy communities. Furthermore, when a shared ESS is installed in a community, as considered in this work, its management can be handled by a utility, cooperative, or third-party operator. Operating costs, e.g., service fees, should be transparently allocated, either equally or based on storage use. The cost structures can include flat rates, usage-based charges, or dynamic allocation. Governance and cost allocation must be clear to ensure acceptance, sustainability, and efficient operation.

The proposed solution also demonstrates the benefits of employing the MARL approach in smart communities. However, its scalability to larger communities or more complex, dynamic environments may be limited by increased computational demands and longer training times. Although the computational complexity of the proposed solution, as designed, increases linearly with the number of agents, coordination and communication overhead can still become significant as the system grows. This may make it more challenging to learn optimal policies and maintain effective collaboration, especially in larger or more heterogeneous communities. Additionally, there is a potential risk of diminishing returns, as the benefits of increasing community size may be offset by the complexity of managing shared resources and ensuring fairness among participants. These trade-offs are common in multi-agent systems and highlight the need for future research on improving scalability and efficiency.

## Conclusion

This paper proposed CoMAD V2G, a MARL-based solution designed to enhance energy utilization in PED with a shared ESS. The proposed solution outperforms conventional approaches and facilitates the achievement of a PED, increasing the energy autonomous of the community by preserving more generated energy in comparison to other more conventional approach. Additionally, CoMAD V2G significantly reduces household electricity costs by up to $25\%$ compared to other heuristic approaches, demonstrating the financial benefits of participating in a community. The findings also show that households within the community can achieve annual savings in the range of 600€, further reinforcing the economic viability of cooperative energy management. Moreover, CoMAD V2G ensures minimal disruption to user comfort through a non-intrusive approach to energy management, effectively balancing the dynamic needs of V2G-enabled EVs, shared batteries, and household energy consumption. These findings underscore the potential of smart, adaptive solutions in advancing sustainable energy management and fostering the growth of collaborative energy communities.

To address the challenges associated with predicting user behavior and managing computational complexity, future work will incorporate advanced forecasting and control methods. Specifically, data-driven models utilizing Long Short-Term Memory (LSTM) neural networks will be developed to predict EV usage patterns, such as departure and arrival times, as well as to improve the accuracy of solar power generation forecasts by capturing temporal dependencies in historical and contextual data, including working hours, weather conditions, and holidays. For system control, advanced reinforcement learning algorithms, for example Proximal Policy Optimization (PPO)^30^, will be employed to dynamically optimize charging and discharging schedules for households and energy exchanges between EVs, households, and the shared ESS, leveraging the aforementioned forecasts. The adaptive nature of PPO enables scalable and fine-grained control that balances individual preferences, user comfort, cost efficiency, and community-wide benefits. To further enhance scalability and address competition among households, Deep W-Networks (DWNs)^31^ will be explored for their effectiveness in managing large multi-agent environments and optimizing distributed energy management tasks. Additionally, the intelligence of the shared ESS will be enhanced by replacing the current static, threshold-based strategy with a learning agent capable of adapting to dynamic electricity prices and predicting overall community consumption, thereby enabling proactive operation that maximizes both user comfort and community benefit.

## Data Availability

The datasets generated during the current study are available in the GitHub repository at github.com/hribarjernej89/CoMAD-V2G. This repository includes the implementation code, links to pre-trained agents, and access to the datasets used in the study.
